# Fetal Limb Ischaemia in Twin-to-Twin Transfusion Syndrome

**DOI:** 10.1155/2013/278726

**Published:** 2013-12-09

**Authors:** Mark Kilby, Rachel Pounds, Paul Mannix

**Affiliations:** ^1^School of Clinical & Experimental Medicine, College of Medical & Dental Sciences, University of Birmingham, Birmingham B15 2TT, UK; ^2^Fetal Medicine Centre, Birmingham Women's Foundation Trust, Edgbaston, Birmingham B15 2TG, UK; ^3^Southmead Hospital, Westbury-on-Trym, Bristol BS10 5NB, UK

## Abstract

*Objective*. To describe the rare association between prenatal vascular limb occlusion and twin-to-twin transfusion syndrome. *The Case*. A woman with severe twin-to-twin transfusion syndrome was treated with fetoscopic laser ablation at 19-week gestation. At 27 weeks, the twins were delivered by an emergency caesarean section. The right arm of twin 1, the recipient twin, was noted to be “ischaemic” and was later amputated. *Conclusion*. This case is unusual in that it affected the upper limb and there was no evidence of polycythaemia, which is a suggested pathological mechanism. It was initially thought that the limb damage was due to the laser ablation, but after discussion with the fetal medicine team vascular limb occlusion in association with twin-to-twin transfusion syndrome was considered. Limb ischaemia is a serious complication of twin-to-twin transfusion syndrome and is unrelated to any form of fetal therapy. *Implications*. Neonatologists and paediatricians need to be aware of this association as it has medicolegal implications and parents should be counselled as to the possible, albeit rare, occurrence, especially when twin-to-twin transfusion syndrome is of advanced stage at presentation.

## 1. Introduction

Twin-to-twin transfusion syndrome (TTTS) occurs in approximately 10% of the monochorionic twin pregnancies. Without treatment, the fetal mortality associated with this morbid prenatal condition is 90%, with over 50% of the survivors having significant morbidity, including neurologic and cardiovascular complications [1].

Although numerous treatment strategies have been advocated, selective fetoscopic laser ablation of placental anastomoses appears to significantly improve the outcome, with the overall outcome increasing to 64% (and at least one survivor to 82%) [2] whilst reducing morbidity.

Prenatal ischaemic limb injury is a rare complication of twin-to-twin transfusion syndrome that appears to occur irrespective of management. Ischaemic limb injury can also occur postnatally in recipient twins, despite being caused by the TTTS in utero. Again, this is unrelated to any interventions [3]. A recent multicentre cohort study from ten centres in North America indicated that limb ischaemia has an incidence of 0.51% and, therefore, in TTTS has a tenfold increasing risk over uncomplicated monochorionic twins [4].

We discuss a single case study of prenatal vascular limb occlusion associated with severe TTTS treated by fetoscopic laser ablation.

## 2. Case Presentation

A 20-year-old multiparous woman (gravida 4, para 3) was referred to a tertiary referral Fetal Medicine Centre at 19 weeks and 3 days with severe twin-to-twin transfusion syndrome (Quintero stage III recipient; III donor) in the UK. The recipient twin had a maximum amniotic pool depth of 10 cm, with intracardiac Doppler evidence of cardiac dysfunction, whilst the donor twin had significant oligohydramnios (maximum pool depth of less than one centimetre), no fetal urine visible in the fetal bladder, and absent end-diastolic velocity on Doppler insonation of the umbilical artery.

After informed consent and counselling, the patient underwent fetoscopic laser ablation. A 2 mm fetoscope was introduced into the recipient twin sac under local anaesthetic and maternal remifentanil sedation. Visualization of the intertwin membrane was achieved and the chorionic plate vasculature was mapped from the recipient and donor cord insertions. Nine arteriovenous anastomoses were visualized and coagulated using a diode laser at power 30 to 40 Watts. The procedure was performed as a sequential selective technique. At the end of the procedure, 2 litres of amniotic fluid was removed by amniodrainage as would be routine in such cases. The procedure was uneventful.

The pregnancy was then monitored by weekly ultrasound scans. No recurrence of the twin oligohydramnios/polyhydramnios sequence was noted and serial measurements of the middle cerebral artery peak systolic velocity were within normal limits for both twins. No fetal anomaly was visualised on fetal ultrasound on followup.

At 26 weeks and 2 days, the patient was admitted to their local District General Hospital with prelabour ruptured membranes confirmed clinically. This was treated conservatively by instituting maternal infection surveillance and prescribing prophylactic antibiotics.

However, at 27 weeks and 4 days, the patient started to have regular contractions and a presumptive diagnosis of preterm labour was made. The patient was transferred to a centre with regional neonatal intensive care cots available, and, on arrival, at 27 weeks and 5 days, the babies were delivered by emergency caesarean section because of “fetal distress.”

**Figure 1:**
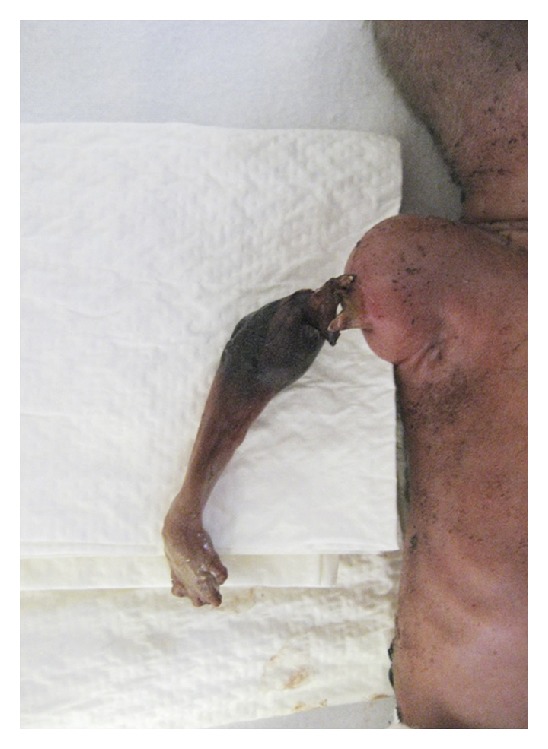
Peripheral vascular ischaemic injury of the right arm at delivery. Fetoscopic laser ablation was performed for severe TTTS seven weeks previously, where no obvious abnormality of the recipient was noted.

Twin I, the recipient twin, weighed 850 grams and had an Apgar score of 8 at one minute and 10 at 5 minutes. He was intubated and ventilated for respiratory distress syndrome and received surfactant. The right arm was noted to be “ischaemic” at the mid-upper arm level (Figure 1). The donor co-twin (Twin II) was delivered weighing 665 grams. Again he was ventilated for respiratory distress syndrome.

The recipient twin had a full blood count test performed in the first few hours of life and this recorded a haemoglobin of 16.8 g/dL with a reticulocyte count of 66 per 100 blood cells. The donor co-twin similarly had recorded haemoglobin of 16.5 g/dL with a reticulocyte count of 180 per 100 blood cells. Initial serum bilirubin levels were also similar with Twin I being 63 micromol/L and Twin II being 48 micromol/L. There was therefore no evidence of twin anaemia/polycythaemia sequence diagnosed. The recipient twin's right arm was amputated by a Consultant Plastic Surgeon under local anaesthetic with parenteral Morphine sedation. This baby had an uncomplicated recovery from this procedure.

Both babies had complications of prematurity but remained well to be discharged to their local District General Hospital and subsequently home.

## 3. Discussion

Vascular limb occlusion associated with twin-to-twin transfusion syndrome is a rare phenomenon. It has an incidence of 0.5%, and to date, in the literature, only 28 cases have been described [4]. Cases have been described in mild twin-to-twin transfusion syndrome that has been managed conservatively [5], associated with serial radical amniodrainage [6] and also noted at or after the treatment by fetoscopic laser ablation [4]. In 90% of the cases, it is the recipient that is affected and mainly affects the lower limbs (80%) [5]. Various pathological mechanisms have been suggested that have included the polycythaemia-hyperviscosity syndrome, release of thrombi after co-twin demise, umbilical arterial-steal syndrome, vascular injury, and elevated levels of vasoconstrictive hormones circulating in the recipient circulation [6].

This case is unusual in that it affected the upper limb of the recipient and clearly there was no documented evidence of polycythaemia in the recipient twin that underwent amputation. As has been described in the literature, this rare complication is more common in advanced Quintero stage disease with the majority of the cases occurring in stage III or IV advanced disease. The neonatal team at the Tertiary unit initially thought that the limb damage was most likely to be due to either an amniotic band disruption following the laser diode treatment or due to a direct effect from the laser ablation itself. It was only after discussion with the fetal medicine team that the information about vascular limb occlusion in association with twin-to-twin transfusion syndrome was considered. Fetal limb ischaemia is therefore a serious complication of twin-to-twin transfusion syndrome and is unrelated to any form of fetal therapy. It has obvious medico-legal implications and parents should be counselled as to the possible, albeit rare, occurrence, especially when twin-to-twin transfusion syndrome is of advanced stage at presentation. Neonatologists and paediatricians working at the neonatal units need to be aware of this association in order for them to be able to discuss this with parents should they have a baby born with this type of condition.
